# Supplementary material for: Targeting SARS-CoV-2 nsp13 Helicase and Assessment of Druggability Pockets: Identification of Two Potent Inhibitors by a Multi-Site In Silico Drug Repurposing Approach
Source: Molecules. 2022 Nov 3;27(21):7522. doi: 10.3390/molecules27217522 (PMC9654784; doi:10.3390/molecules27217522)

Supplementary Material for

Targeting SARS-CoV-2 nsp13 helicase and assessment of druggability pockets: identification of two potent inhibitors by a multi-site *in silico* drug repurposing approach

Isabella Romeo,[§,†] Francesca Alessandra Ambrosio,[±] Giosuè Costa,[§,†] Angela Corona,[‡] Mohammad Alkhatib,[§] Romina Salpini,[§] Saverio Lemme,[§] Davide Vergni,[Ψ] Valentina Svicher,[§] Maria Mercedes Santoro,[§] Enzo Tramontano,[‡] Francesca Ceccherini-Silberstein[§], Anna Artese[§,†,*] and Stefano Alcaro[§,†]

[§]Dipartimento di Scienze della Salute, Università degli Studi "Magna Græcia" di Catanzaro, Campus "S. Venuta", Viale Europa, 88100 Catanzaro, Italy;

[†]Net4Science Academic Spin-Off, Università degli Studi "Magna Græcia" di Catanzaro, Campus "S. Venuta", Viale Europa, 88100 Catanzaro, Italy;

[±]Dipartimento di Medicina Sperimentale e Clinica, Università degli Studi "Magna Græcia" di Catanzaro, Campus "S. Venuta", Viale Europa, 88100 Catanzaro, Italy;

[‡]Department of Life and Environmental Sciences, University of Cagliari, Cittadella Universitaria di Monserrato, 09124 Cagliari, Italy;

[§]Dipartimento di Medicina Sperimentale, Università Tor Vergata di Roma, Via Montpellier, 1, 00133 Roma, Italy;

[Ψ]Istituto per le Applicazioni del Calcolo "Mauro Picone" - CNR, Rome, Italy.

Supplementary Figure S1

Supplementary Table S1

Supplementary Figure S2

Supplementary Figure S3

Supplementary Figure S4

Supplementary Figure S5

Supplementary Figure S6

Supplementary Figure S7

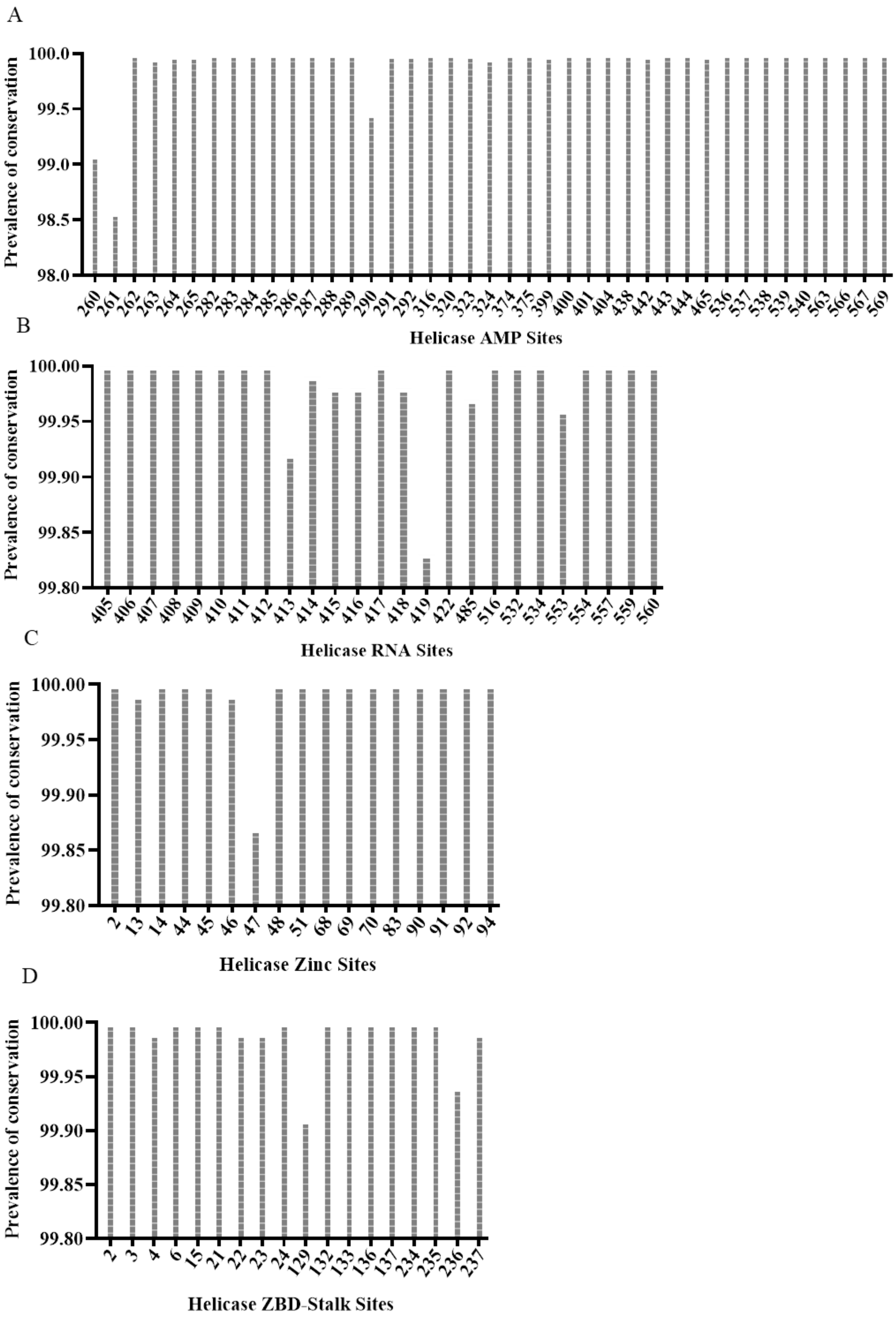

Figure S1. The histograms report the degree of genetic conservation of the key residues in SARS-CoV-2 helicase functional regions: (**A**) AMP sites (Pocket 1), (**B**) RNA sites (Pocket 2), (**C**) Zinc sites (Pocket 4), and (**D**) ZBD-Stalk sites (Pocket 3).

Table S1. Residues for the grid generation of each pocket of SARS-CoV-2 nsp13.

Pocket	Residues
**1**	Asp260-Asn265; Gly282-Ala292; Ala316; Lys320; Lys323; Tyr324; Asp374; Glu375; Ile399-Asp401; Gln404; Leu438; Arg442-Cys444; Lys465; Ser536-Glu540; Val563; Thr566; Arg567;Lys569
**2**	Leu405-Pro419; Phe422; Ser485; Asn516; Thr532; Asp534; Ala553; Asn557; Asn559; Arg560
**3**	Val2-Ala4; Val6; Arg15; Arg21-Phe24; Arg129; Leu132; Phe133; Glu136; Thr137; Pro234-Ala237
**4**	Ser13; Leu14; Ser44-Tyr48; Asn51; Met68-Tyr70; Leu83; Phe90-Leu92; Lys94

Figure S2. 3D representation of the best docking pose of (**A**) acteoside, (**B**) cefpiramide, (**C**) ceftaroline fosamil, (**D**) metaraminol, (**E**) inarigivir soproxil, (**F**) NADH, (**G**) riboflavin, (**H**) PF-00610355, (**I**) polydatin, (**J**) rutin, (**K**) regadenoson, (**L**) PF-03715455 into the SARS-CoV-2 nsp13 pocket 2.

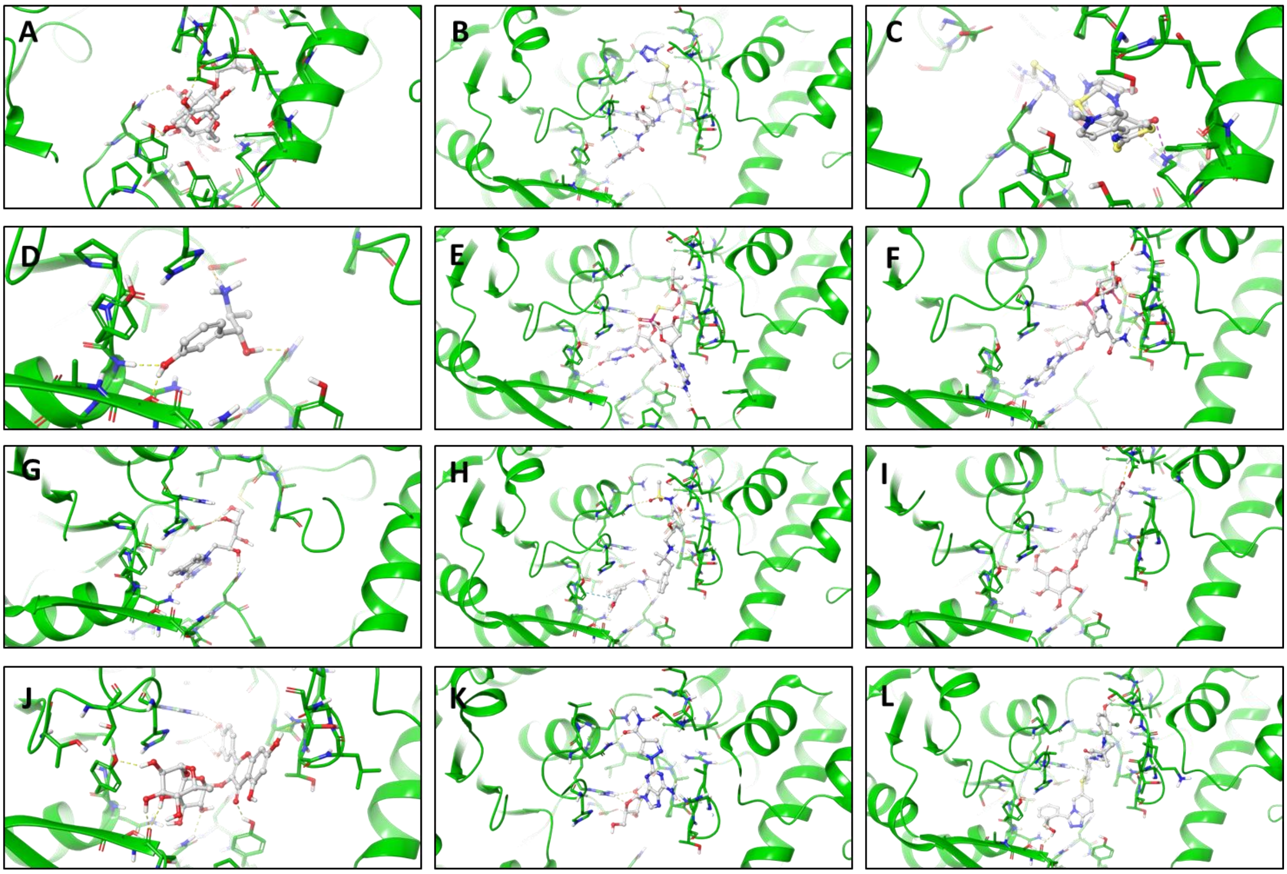

Figure S3. 3D representation of the best docking pose of (**A**) ceftaroline fosamil, (**B**) polydatin, (**C**) PF-00610355, (**D**) inarigivir soproxil, (**E**) NADH, (**F**) PF-03715455, (**G**) rutin, (**H**) acteoside, (**I**) metaraminol, (**J**) regadenoson, (**K**) 5-methyltetrahydrofolic acid, (**L**) foscarnet, (**M**) riboflavin, (**N**) cefpiramide into the SARS-CoV-2 nsp13 pocket 3.

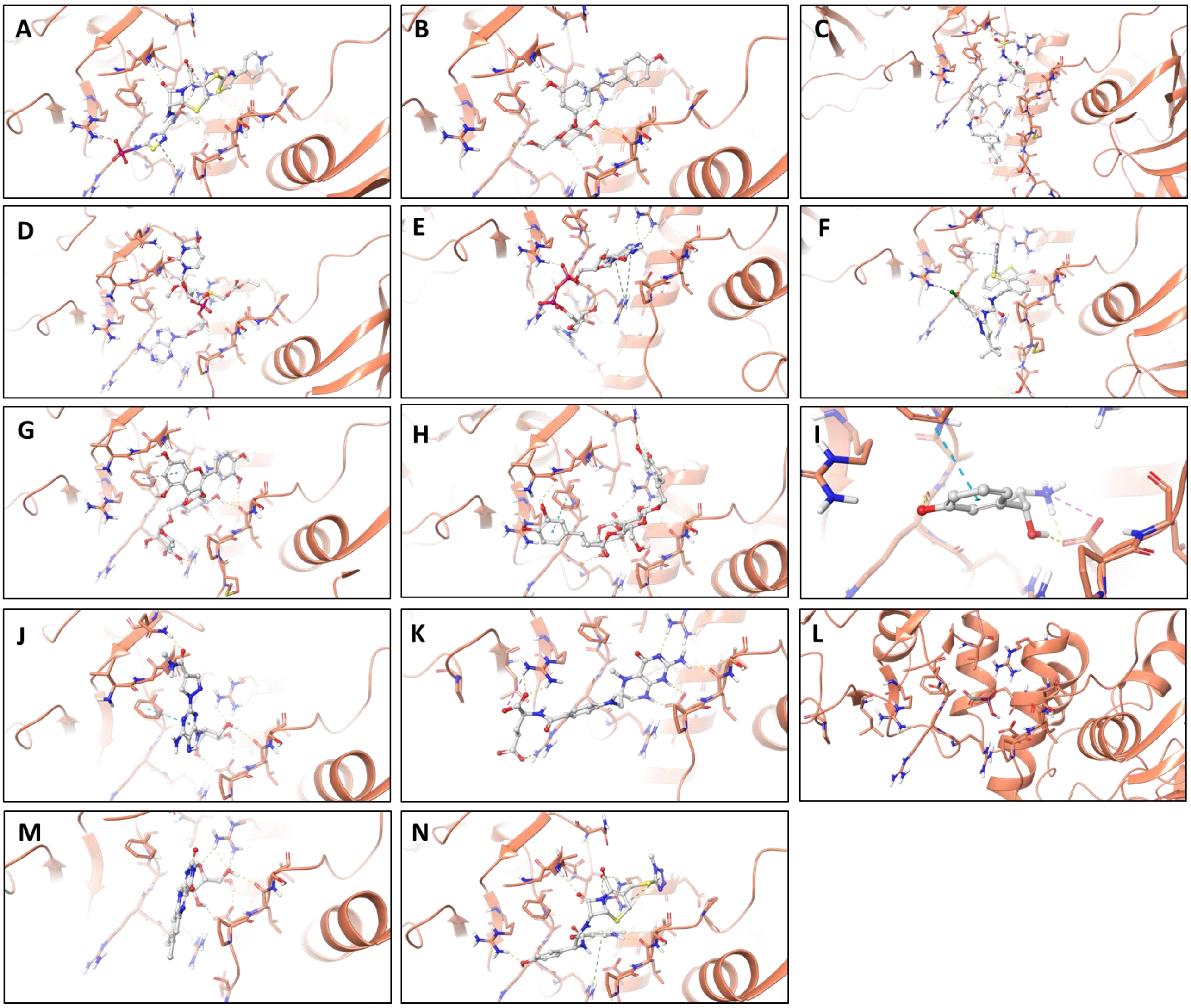

Figure S4. 3D representation of the best docking pose of (**A**) acteoside, (**B**) rutin, (**C**) metaraminol, (**D**) ceftaroline fosamil, (**E**) inarigivir soproxil, (**F**) PF-03715455, (**G**) foscarnet, (**H**) polydatin, (**I**) regadenoson, (**J**) NADH, (**K**) riboflavin, (**L**) PF-00610355, (**M**) cefpiramide, (**N**) 5-methyltetrahydrofolic acid into the SARS-CoV-2 nsp13 pocket 4.

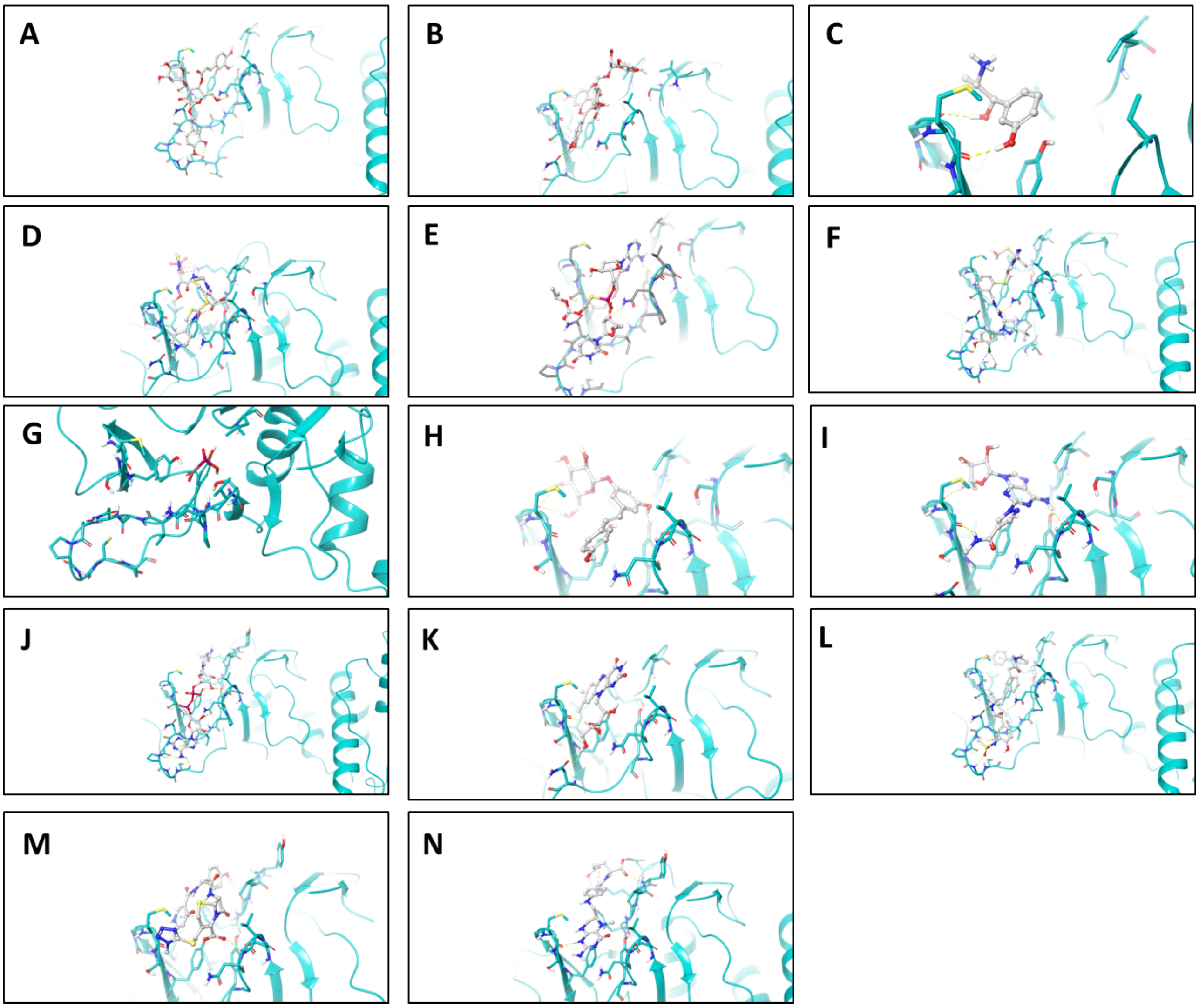

Figure S5. 2D representation of the best docking pose of (**A**) PF-00610355, (**B**) PF-03715455, (**C**) NADH, (**D**) ceftaroline fosamil and (**E**) polydatin into the SARS-CoV-2 nsp13 pocket 2.

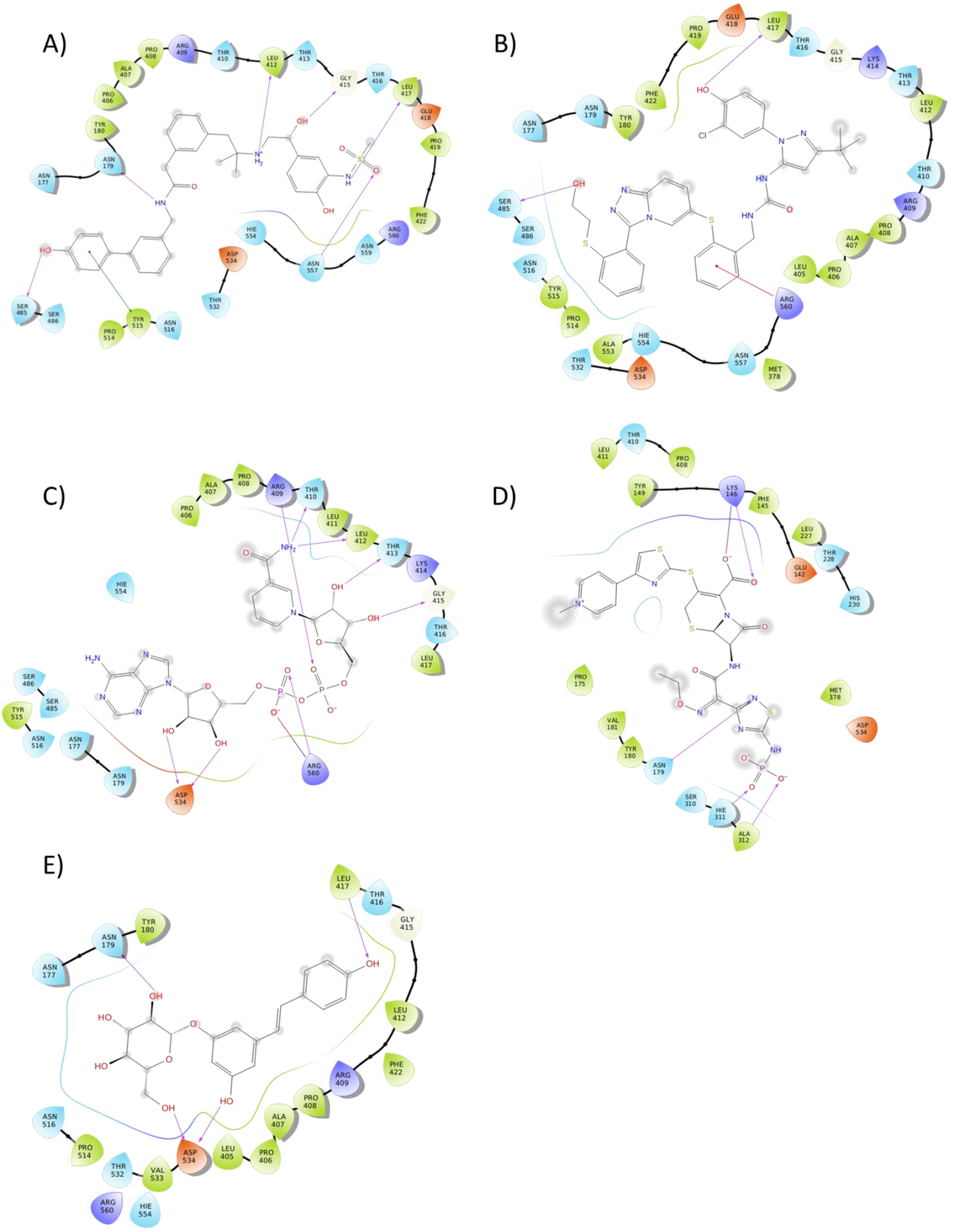

Figure S6. 2D representation of the best docking pose of (**A**) PF-00610355, (**B**) PF-03715455, (**C**) NADH, (**D**) ceftaroline fosamil and (**E**) polydatin into the SARS-CoV-2 nsp13 pocket 3.

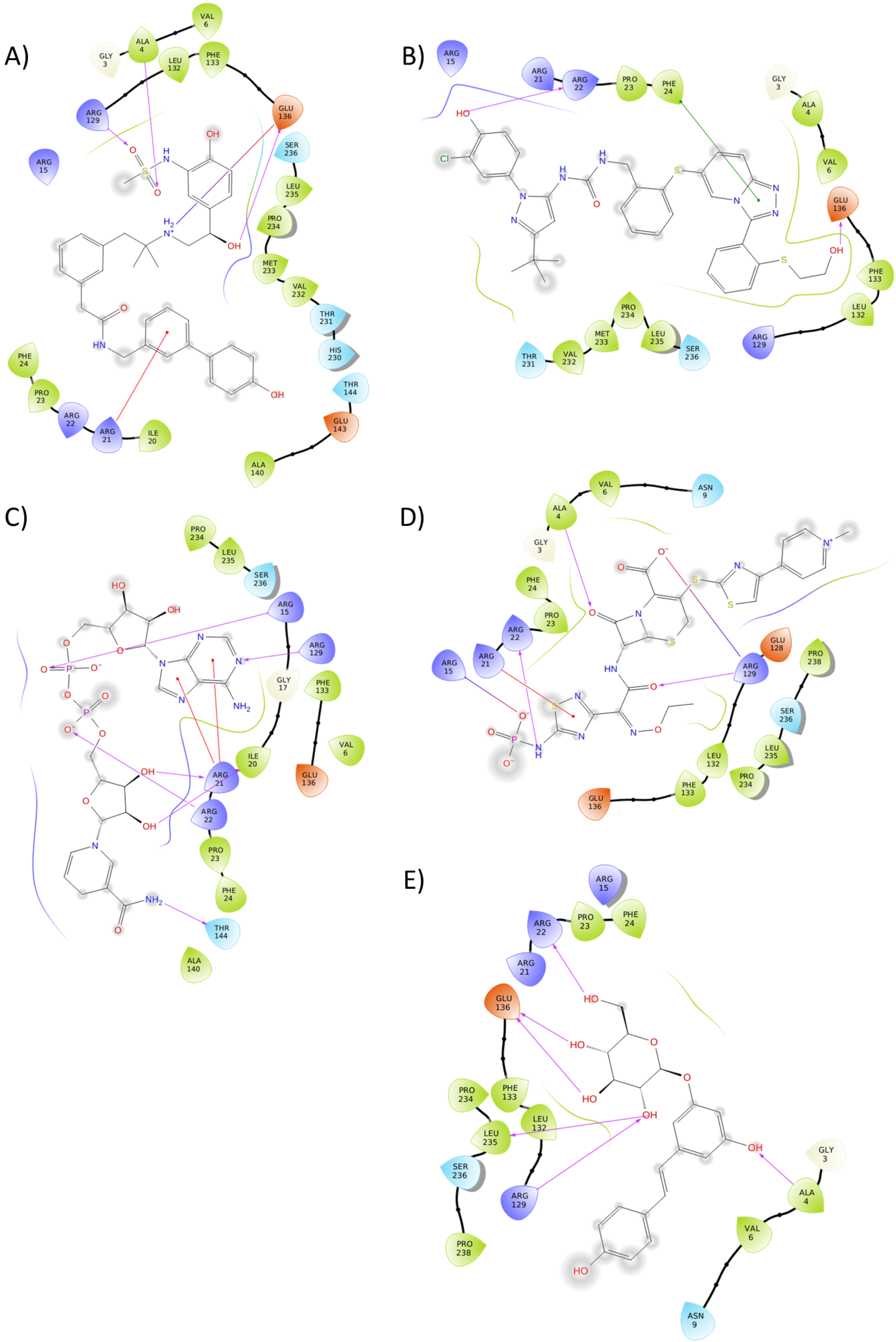

Figure S7. 2D representation of the best docking pose of (**A**) PF-00610355, (**B**) PF-03715455, (**C**) NADH, (**D**) ceftaroline fosamil and (**E**) polydatin into the SARS-CoV-2 nsp13 pocket 4.

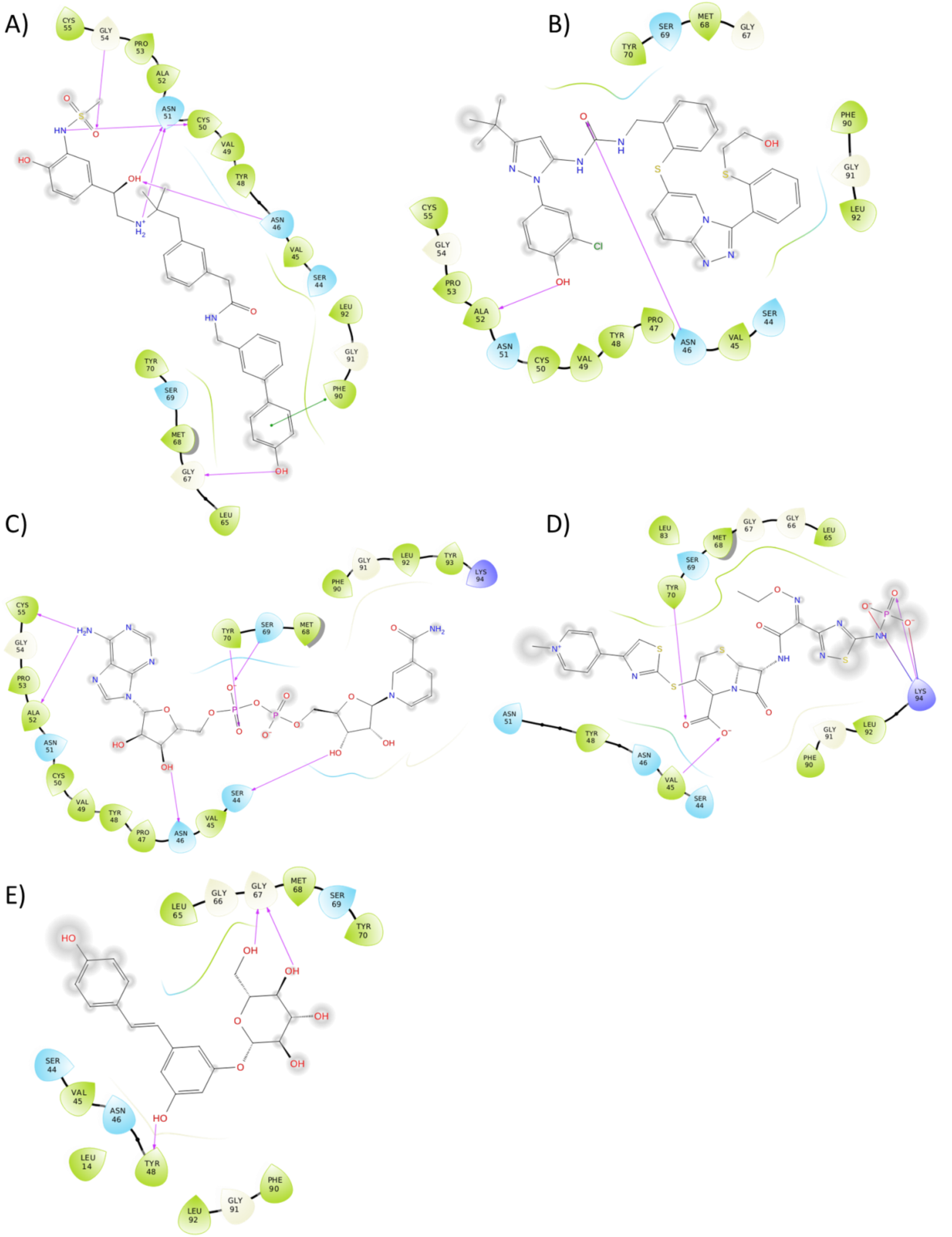

Supplement: Supplementary file 1 [file molecules-27-07522-s001.zip › molecules-1951720-supplementary.pdf]
